# Phenotypic and functional analyses of NK and NKT-like populations during the early stages of chikungunya infection

**DOI:** 10.3389/fmicb.2015.00895

**Published:** 2015-09-01

**Authors:** Subrat Thanapati, Rumki Das, Anuradha S. Tripathy

**Affiliations:** Hepatitis Group, National Institute of VirologyPune, India

**Keywords:** chikungunya, NK cell, NKT-like cell, cytotoxicity, perforin

## Abstract

The aim of this study was to characterize NK (CD56^+^CD3^−^) and NKT-like cell (CD56^+^CD3^+^) responses early after chikungunya infection. Expression profiling and functional analysis of T/NK/NKT-like cells were performed on samples from 56 acute and 31 convalescent chikungunya patients and 56 control individuals. The percentages of NK cells were high in both patient groups, whereas NKT-like cell percentages were high only in the convalescent group. The percentages of NKp30^+^CD3^−^CD56^+^, NKp30^+^CD3^+^CD56^+^, CD244^+^CD3^−^CD56^+^, and CD244^+^CD3^+^CD56^+^cells were high, whereas the percentages of NKG2D^+^CD3^−^CD56^+^ and NKG2D^+^CD3^+^CD56^+^cells were low in both patient groups. The percentages of NKp44^+^CD3^−^CD56^+^ cells were high in both patient groups, whereas the percentages of NKp44^+^CD3^+^CD56^+^ cells were higher in the acute group than in convalescent and control groups. The percentages of NKp46^+^CD3^−^CD56^+^ cells were high in both patient groups. Higher percentages of perforin^+^CD3^−^CD56^+^ and perforin^+^CD3^+^CD56^+^ cells were observed in acute and convalescent patients, respectively. Higher cytotoxic activity was observed in acute patients than in controls. IFN-γ expression on NK cells of convalescent patients and on NKT-like cells of both patient groups was indicative of the regulatory role of NK and NKT-like cells. Collectively, these data showed that higher expression of activating receptors on NK/NKT-like cells and perforin^+^ NK cells in acute patients could be responsible for increased cytotoxicity. The observed expression of perforin^+^ NK cells in the acute phase and IFN-γ^+^ NKT-like cells in the subsequent convalescent stage showed that NK/NKT-like cells mount an early and efficient response to chikungunya virus. Further study of the molecular mechanisms that limit viral dissemination/establishment of chronic disease will aid in understanding how NK/NKT-like cells control chikungunya infection.

## Introduction

Chikungunya (CHIK), a mosquito-borne viral disease caused by the chikungunya virus (CHIKV), has caused global concern since its re-emergence in several Asian and African countries (Powers and Logue, 2007). CHIKV belongs to the *Alphavirus* genus of the family Togaviridae, and is responsible for severe rheumatic manifestations associated with inflammation and musculoskeletal tissue destruction in humans (Suhrbier and La Linn, 2004). CHIK epidemics have recently been reported in new areas, such as North America and the Caribbean, where the populations are naïve to this viral infection (Cassadou et al., 2013; Leparc-Goffart et al., 2014; Van Bortel et al., 2014). Over 3 million suspected cases of CHIK have been recorded worldwide till now (Seppa, 2015). In the absence of a specific treatment, recent epidemics in previously naïve parts of the world have elevated CHIK to a global health problem. The symptoms of CHIK appear after an incubation period of 4−7 days following CHIKV infection and mostly resolve within the acute phase. Although, the acute phase lasts for approximately 2 weeks, joint pain can persist for months or years following the initial infection, which is the hallmark of chronic CHIKV infection (Kelvin et al., 2011; Dupuis-Maguiraga et al., 2012). Recently reported CHIK outbreaks have shown severe haemorrhagic and neurologic manifestations, which could be attributed to host immune response (Sissoko et al., 2008; Suhrbier et al., 2012). The innate immune response plays a key role in virus suppression, propagation, and dissemination before induction of the adaptive immune response. Natural killer (NK) and natural killer T (NKT) cells can kill target cells directly or interact with antigen-presenting cells, T cells to produce cytokines, which have antiviral activities and can trigger an adaptive immune response (Janeway and Medzhitov, 2002). NKT-like cells are a subset of αβ T cells that express NK activation receptors and also exhibit a highly specialized effector memory phenotype (Peralbo et al., 2007; Tang et al., 2013). The functional activity of NK/NKT-like cells is regulated through their repertoire of activation (NKG2C, NKG2D, NKp30, NKp44, and NKp46) and inhibitory (CD158a, CD158b, KIR3DL1, and NKG2A) receptors, which recognize ligands on the surface of target cells (Peralbo et al., 2007; Watzl and Long, 2010; Das and Tripathy, 2014). Upon activation, both NK and NKT-like cells produce inflammatory cytokines, such as IFN-γ, and lyse target cells by exocytosis of perforin and granzyme, leading to inhibition of viral replication and enhancement of cytotoxicity against target cells (Biron and Brossay, 2001; Janeway and Medzhitov, 2002; Peralbo et al., 2007; Das and Tripathy, 2014). During the granule dependent mechanism for killing of target cells, the lysosomal membrane associated protein 1 (LAMP1/CD107a) becomes detectable on the surface of NK cells and CTLs, indicating that CD107a expression is a marker of degranulation (Kannan et al., 1996; Bossi and Griffiths, 1999). NK cells contribution toward arthritis in the Ross River virus infection has been reported by Aaskov et al. (1987).

The higher levels of IFN-α and IL-12 observed in monocyte cultures within 2 h of CHIKV infection suggested their possible involvement in activating NK cells to increase antiviral activities (Gherardi et al., 2003; Wang et al., 2009; Her et al., 2010). Studies on humans and non-human primates have shown increased NK cell numbers in the early stages of CHIKV infection and suggested their participation in the early control of CHIKV (Labadie et al., 2010; Watzl and Long, 2010). Petitdemange et al. (2011) performed phenotypic and functional analyses of NK cells from 25 patients in the early stages of acute CHIKV infection, and showed engagement of a clonal expansion of CD94/NKG2C NK cells that expressed receptors for HLA-C1 alleles, and correlated with the viral load, suggesting that NK cells sense CHIKV from the beginning of infection and may thus contribute to viral clearance (Petitdemange et al., 2011). In contrast, a study of CHIKV infection in knockout mice indicated that T cells but not NK cells play a role in viremia suppression (Poo et al., 2014). However, the available literature on NK cells in CHIKV infection is limited.

In the current study, we performed peripheral phenotypic and functional analyses of T/NK/NKT-like cells from patients in the acute and convalescent stages of CHIKV infection, to assess the role of these lymphocytes in CHIK pathogenesis.

## Materials and methods

### Study population

The study population comprised 87 patients from the CHIK outbreak of September 2011 in Maharashtra, India, and 56 healthy controls from blood donation camps in Maharashtra. The diagnosis of CHIKV was based on the presence of IgM antibodies against the virus (anti-CHIKV IgM) as determined by ELISA and/or RT-PCR analysis of CHIKV RNA. The control group consisted of age- and sex-matched healthy individuals negative for anti-CHIKV IgM/IgG antibodies. Based on the number of days post-onset of illness (POD), patients were categorized as acute (POD≤14) or convalescent (POD>14) (Table 1). The patient groups were comprised of 56 acute and 31 convalescent patients. The gender ratios (male: female) in the study groups were as follows: 1.80 for acute, 0.29 for convalescent, and 1.54 for controls. The convalescent patients were symptomatic at the time of enrolment and were CHIK RNA-negative. The study was approved by the Institutional Ethical Committee for Research on Humans, based on the guidelines set by the Indian Council of Medical Research, New Delhi. Informed written consent was obtained from all participants.

**Table 1:** **Characteristics of study population**.

**Parameters**	**Patients**		**Controls**
	**Acute**	**Convalescent**	
Study population	*n* = 56	*n* = 31	*n* = 56
Sex ratio (Male:Female)	1.80	0.29	1.54
Age (years): median (range)	38 (13−70)	40(18−82)	27(18−47)
POD: median (range)	12 (2−14)	33 (16−80)	NA
Anti-CHIKV IgM	Positive, *n* = 46	Positive	Negative
Anti-CHIKV IgG	Positive, *n* = 32	Positive	Negative
Viral load(copies/ml): median (range)	110 (3100−670000000), *n* = 23	ND	NA

*NA, not applicable; ND, not detected; POD, post onset days of illness*.

### Serological testing

All samples were screened by ELISA for anti-CHIKV IgM and IgG antibodies and for antibodies against dengue virus (Yergolkar et al., 2006). Only samples that were negative for dengue virus were included in the study.

### Viral load quantification

Plasma samples from patients in the acute phase were processed for quantitation of CHIKV RNA. Briefly, RNA was extracted using QIAamp viral RNA mini kit (Qiagen, Hilden, Germany) and the viral load (copies/ml) was determined using primers and probes based on CHIKV E3, according to a previously described protocol (Patil et al., 2012).

### CHIKV purification and inactivation by β-Propiolactone (BPL)

The CHIKV strain (accession number EF027134) used in the current study was obtained from the virus repository of the National Institute of Virology, Pune, India. Following incubation in the Vero E6 cell line, CHIKV was harvested when cells exhibited 80−90% cytopathic effect. The clarified supernatant was removed, lysed cell pellets were centrifuged, and the supernatant was pooled with the culture supernatant. CHIKV was purified and was subsequently inactivated as previously described (Kumar et al., 2012). Following protein estimation, whole, inactivated virus was used as the antigen in the ELISPOT assay.

### Flow cytometric analysis

#### CD3^+^, CD3^+^CD4^+^, and CD3^+^CD8^+^ T cell enumeration

Freshly drawn peripheral blood samples (100 μl) from the 45 acute and 31 convalescent patients and the 36 controls were stained with anti-human CD3 (clone SK7), CD4 (clone RPA-T4), and CD8 (clone RPA-T8) monoclonal antibodies (BD Biosciences, CA, USA) as previously described (Tripathy et al., 2011). The lymphocyte population was distinguished on the basis of their forward and side scatter properties. Within the lymphocyte population, the CD3^+^ T cell population was gated based on the CD3 staining pattern, whereas the CD3^+^CD4^+^and CD3^+^CD8^+^ cells were gated on CD3^+^ T cells. For each experiment, 50,000 events were acquired within the lymphocyte gate and results are expressed as the percentage of positive cells in the gated population.

#### NK/NKT-like cells and their receptors

Enumeration of NK/NKT-like cells and expression of activation and inhibitory receptors were investigated in samples from 56 acute and 31 convalescent patients and 56 control individuals. CD161 and inhibitory receptors were assessed only in acute patients and controls. Peripheral blood (100 μl) was stained according to a previously described protocol (Tripathy et al., 2011). The monoclonal antibodies used were anti-humanCD56 (clone B159), CD3 (clone SK7/UCHT1), NKp30/NKp44/NKp46/CD244/CD161/NKG2D (clone p30-15/p44-8/9E2/2-69/DX12/1D11), purchased from BD Biosciences and CD94 (clone 131412 APC), and NKG2A (clone 131411) purchased from R&D Systems Inc., MN, USA. NK (CD56^+^CD3^−^) and NKT-like (CD56^+^CD3^+^) cells were gated within the lymphocyte population on the basis of their CD56 and CD3 staining pattern; their receptors expression patterns are expressed as the percentage of gated NK and NKT-like cells.

#### CFSE-based cytotoxicity assay

A carboxyfluorescein succinimidyl ester (CFSE; Invitrogen, NY, USA)-based cytotoxicity assay was performed in samples from eight acute patients, six convalescent patients, and eight healthy controls following a previously described method (Fu et al., 2010; Das and Tripathy, 2014). Briefly, K562 cells (Target, T) were labeled with CFSE (0.2μM/10^6^ K562 cells). The cells were washed and plated in 96-well U-bottom plates and fresh PBMCs (Effector, E) were added to the target cells at a 10:1 E:T ratio. This was followed by incubation at 37°C with 5% CO_2_ for 6 h. Target cells treated with 0.1% Triton-X (Sigma, MO, USA) and unlabeled effector/target cells served as controls. To assess cell lysis, tubes were kept on ice with 2 μg/ml of 7-AAD (BD Biosciences) and incubated for 5 min to label the DNA of the dead cells. This was followed by immediate acquisition in a BD FACS ARIA II. During acquisition, 5000 target events were recorded. CFSE^+^/7-AAD^+^ cells were considered to be dead target cells. The readings for CFSE^+^/7AAD^+^cells of target cells were subtracted from the CFSE^+^/7-AAD^+^ cells of the E/T cells for normalization.

%specific killing = [(%experimental target death - %spontaneous target death)/(100 - %spontaneous target death)] × 100.

#### Degranulation assay

Expression of the CD107a marker on NK/NKT-like cells was quantitated in samples from eight acute patients, six convalescent patients, and eight healthy controls to assess degranulation activity, as previously reported (Alter et al., 2004; Das and Tripathy, 2014). Briefly, PBMCs suspended in RPMI (Invitrogen, NY, USA) ^+^ 10% FBS [effector (E)] were stimulated with MHC I-devoid K562 cells (ATCC, VA, USA) [target (T)] at a 10:1 (E:T) ratio along with anti-CD107a (Lamp1)(clone H4A3) antibody (BD Biosciences) at 37°C. Culture medium [RPMI medium supplemented with 10% FBS] alone and PMA (0.25 μg/ml) + ionomycin (0.5 μg/ml) (Sigma, MO, USA)-treated PBMCs served as negative and positive controls, respectively. Following 1 h of stimulation, 4 μg/ml of brefeldin A (Sigma, MO, USA) and 5 μg/ml of monensin sodium salt (Sigma, MO, USA) were added and the cells were incubated for a total of 6 h. Following incubation, the cells were stained with an anti-human CD56 (clone B159) and CD3 (clone SK7) antibody (BD Biosciences) cocktail, then washed and fixed in 1% paraformaldehyde. Cells were gated on NK and NKT-like cells and the CD107a frequency was evaluated. During acquisition, 5000 events were acquired within the lymphocyte gate. Data were normalized by subtracting the readings of unstimulated PBMCs from PBMCs stimulated with K562 cells.

#### Intracellular cytokines and perforin assays

Intracellular cytokine staining and perforin assays were performed in samples from eight acute patients, six convalescent patients, and eight healthy controls, as described previously (Fu et al., 2010; Das and Tripathy, 2014). Following co-incubation (effector/target) for 6 h, cells were stained with anti-human CD56 (clone B159) and CD3 (clone SK7) monoclonal antibodies (BD Biosciences) to enumerate the respective cell surface antigens. The CD3 and CD56 surface-stained cells were permeabilized for intracellular staining. The monoclonal antibodies, i.e., IFN-γ (clone 25723.11)/TNF-α (clone MAb11)/ perforin (clone δG9) (BD Biosciences) were added to the permeabilized cells, incubated, washed in BD Perm/Wash buffer (BD Biosciences) and fixed in paraformaldehyde. Cells were gated on NK and NKT-like cells. During acquisition, 5000 events were acquired within the lymphocyte gate.

Acquisition and analysis of the samples was performed in BD FACS ARIA II using the FACS DIVA software (BD Biosciences).

### IFN-γ elispot assay

A CHIKV-specific ELISPOT assay for IFN-γ release was performed on samples from 13 acute and 15 convalescent patients and was compared with 18 controls, as previously reported (Tripathy et al., 2011, 2012). PBMCs were isolated as previously reported (Tripathy et al., 2011). To estimate the number of CHIKV-specific IFN-γ-secreting spot-forming cells (SFCs), 1 × 10^5^ PBMCs/well were stimulated with whole purified and inactivated CHIKV (10 μg/ml). In the positive/negative control wells, cells were stimulated with 10 μg/ml of phytohemagglutinin A (Sigma)/medium alone. SFCs were counted in an ELISPOT reader (Carl Zeiss, Jena, Germany) using the KS ELISPOT software and were expressed as the number per 10^5^ cells. The cut-off level for SFCs was set as the average number of SFCs in the negative control wells. Results with high background readings or with no PHA response were excluded. The number of SFCs in unstimulated wells was subtracted from the number in antigen-stimulated wells in each subject category for comparison.

### Software and statistical analysis

Statistical analyses were performed using the SPSS 20 software (SPSS Inc., IL, USA). Intergroup comparisons were assessed using a nonparametric Mann–Whitney U-test (where the difference in variances < 4) and a Kolmogorov–Smirnov test (where the difference in variances >4). The data are expressed as means (range). *P* < 0.05 were considered statistically significant. Spearman's rank correlation was used to assess the correlation between NK/NKT-like cells and NKRs with viral load. Only significant results are shown.

## Results

### NK (CD56^+^CD3^−^) and NKT-like cell (CD56^+^CD3^+^) subsets in CHIK patients

To evaluate the impact of CHIKV infection on the phenotype of innate immune cells, the percentages of NK and NKT-like cells from acute and convalescent patients were assessed using flow cytometry. Higher percentages of NK cells were observed in both patient groups than in controls [acute: 9.4(0.7−29.2) vs. controls: 8.2(1.4−37.6), *p* < 0.001 and convalescent: 9.4(3.6−24.6) vs. controls: 8.2(1.4−37.6), *p* = 0.001] (**Figure 2i**). NK cells in the acute CHIK patients showed a higher absolute count than in controls [acute: 233(158−270) and controls: 175(101−360), per mm^3^] (Supplementary Table 1). The percentage of NKT-like cells was higher in convalescent patients than in controls [convalescent: 17.6(0.3−36.6) vs. controls: 7.3(0.15−26.14), *p* < 0.001] (**Figure 2ii**). Among the patient categories, the percentage of NKT-like cells was higher in convalescent patients [convalescent: 16.7 (0.3−36.6) vs. acute: 10.2 (0.4−27.2), *p* = 0.016] (**Figure 2ii**). Gating strategies for NK and NKT-like cell enumeration are shown in **Figure 2iv**.

The correlation between the percentage of NK cells and the CHIKV load was positive (*r* = 0.605, *p* = 0.004) (**Figure 2iii**).

**Figure 1:**
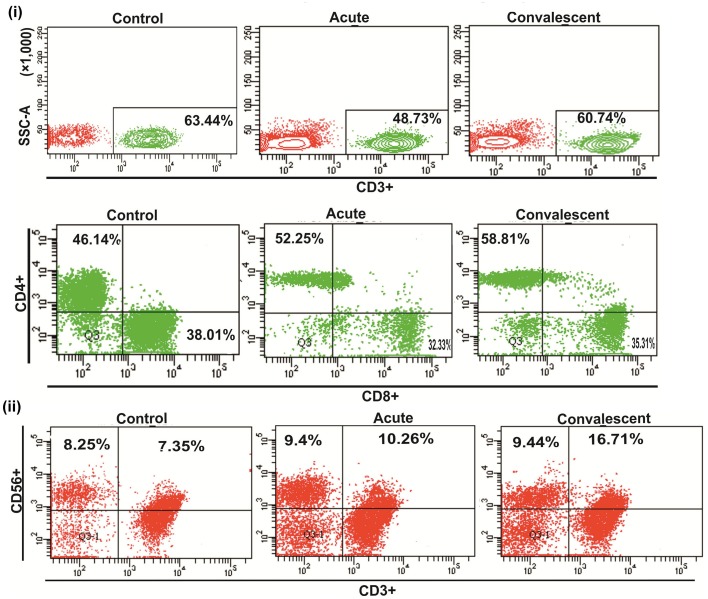
**Percentge of lymphocytes popultion**. (i) Upper pannel shows the representative figures for CD3^+^ T cells proportion in control individuals, acute and convalescent patients, lower pannel shows representative figures for CD3^+^CD4^+^/CD3^+^CD8^+^ T cells proportion in control individuals, acute and convalescent patients. (ii) Shows the representative figures for NK (CD3^−^CD56^+^)/NKT-like(CD3^+^CD56^+^) cells proportion in control individuals, acute and convalescent patients.

Representative figures showing the proportion of NK and NKT-like cells are depicted in (Figure 1ii).

### NK cell receptor (NKR) expression on NK cells from CHIK patients

**Figure 2:**
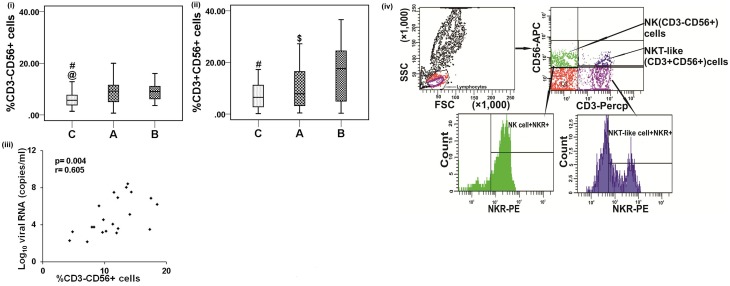
**Distribution of NK and NKT-like cells**. Percentages of the lymphocytes mentioned were determined from the whole blood of 56 control subjects, 56 acute, and 31 convalescent patients. Box plots show the percentages of (i) NK (CD3^−^CD56^+^) cells and (ii) NKT-like (CD3^+^CD56^+^) cells. Boxes represent interquartile ranges, vertical lines represent ranges and horizontal lines represent medians. Nonparametric Kolmogorov–Smirnov test was used for intergroup comparison. @ denotes A (acute) vs. C(control), # denotes B (convalescent) vs. C (control) and $ denotes A (acute) vs.C (convalescent). *p* < 0.05 is considered significant. (iii) Spearman correlation co-efficient shows positive correlation between viral load and NK (CD3^−^CD56^+^) cells from acute patients, where *p* < 0.05 is considered significant and *r* > 0.5 is considered as positive. (iv) Plots showing the strategy used for gating lymphocyte population, NK and NKT-like cells. Lymphocytes are gated in FSC vs. SSC plot, upper left quadrant (Q1) of contour plot shows NK cells (CD3^−^CD56^+^) and upper right quadrant (Q2) shows NKT-like cells (CD56^+^CD3^+^). NK/NKT-like cells were gated within the lymphocytes as per CD56 and CD3 staining pattern and NKRs were gated on NK/NKT-like cells individually. Histogram plots represent the percentage of NKRs^+^ (NK receptors) NK/NKT-like cells.

**Figure 3:**
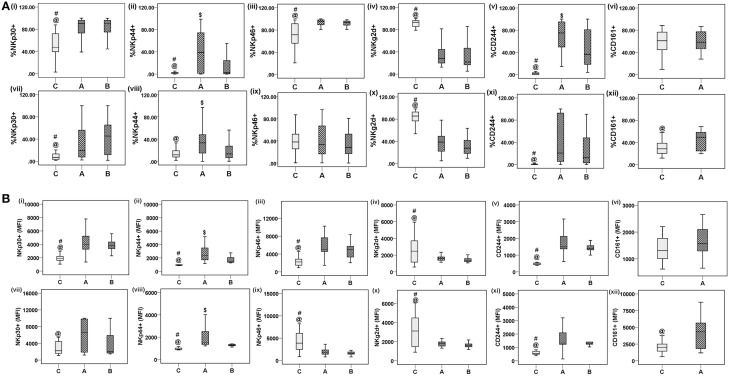
**Patterns of NK receptors (NKRs) on NK and NKT-like cells**. Frequencies of NKRs were enumerated from the whole blood of 30 control subjects, 30 acute, and 31 convalescent patients. **(A)** Box plots (i-vi) show percentages of NKRs^+^ NK (CD3^−^CD56^+^) cells as (i) NKp30, (ii) NKp44, (iii) NKp46, (iv) NKG2D, (v) CD244, (vi) CD161. Box plots (vii–xii) shows FACS analysis of percentages of NKRs^+^ NKT-like (CD3^+^CD56^+^) cells as (vii) NKp30, (viii) NKp44, (ix) NKp46, (x) NKG2D, (xi) CD244, (xii) CD161. CD161 percentage was enumerated in acute patients and controls. **(B)** Box plots show mean fluorescence intensity (MFI) of NKRs^+^ NK cells (i–vi) and NKRs^+^ NKT-like cells (vii–xii). Kolmogorov–Smirnov test was used for intergroup comparison. *p* < 0.05 is considered significant.

Next, we investigated whether the increased NK cell percentages were related to changes in expression of activation and inhibitory receptors. Percentages of NKp30^+^ NK cells were high in acute and convalescent patients [acute: 78.72 (0.9−100), convalescent: 80.3 (5.8−99.8) vs. control: 52.3 (2.9−87.8), *p* = 0.001 for each] (**Figure 3Ai**). The percentage of NK cells bearing NKp44^+^ receptors was high in acute and convalescent patients [acute: 42 (0−98.9), convalescent: 14.8 (0−96.4) vs. controls: 6.8 (0−5.5), *p* = 0.025]. The percentage of NKp44^+^ NK cells was higher in acute than in convalescent patients (*p* = 0.008) (**Figure 3Aii**). The percentage of NKp46^+^ NK cells was high in both patient groups compared to controls [acute: 88.69 (6−100), convalescent: 90.5 (67.3−98.5) vs. controls: 71.4 (0−98.1), *p* = 0.002 for each] (**Figure 3Aiii**). The percentages of CD244^+^ NK cells were higher in both patient categories than in controls [acute: 70.2 (14.8−100), convalescent: 47.8 (4−100) vs. controls: 3 (0.1−28.5), *p* < 0.001 for each]. The percentage of CD244^+^ NK cells was higher in acute than in convalescent patients (*p* = 0.019) (**Figure 3Av**). In the same line the mean fluorescence intensity (MFI) of NKp30^+^, NKp44^+^, NKp46^+^, and CD244^+^ NK cells was also high in patient categories than in controls (**Figures 3Bi−iii, v**). Among the patient categories, MFI of NKp44^+^ NK cells was higher in acute (**Figure 3Bii**). Lower percentages of NK cells bearing NKG2D^+^ receptors were observed in both patient categories compared to controls [acute: 34.3 (12.6−87.5), convalescent: 32.27 (5.2−85.9)] vs. controls: 83.7 (9.5−100), *p* < 0.001 for each] (**Figure 3Aiv**). In consistent with the percentage of NKG2D^+^ NK cells, MFI was also lower in patient categories compared to controls (**Figure 3Biv**). The gating strategy used for enumeration of NKRs is shown in Figure 2iv.

### NKRs on NKT-like cells

The relationship between NKT-like cells and changes in expression of activation and inhibitory receptors was investigated. Higher percentages of NKT-like cells bearing NKp30^+^ receptors were observed in acute and convalescent patients [acute: 33.8 (2.3−100), convalescent: 42.8 (1.8−100) vs. controls: 13.5 (1.6−88.1), *p* = 0.014 for each] (Figure 3Avii). The MFI of NKp30^+^ NKT-like cells were high in acute patients than in controls, however, comparable among convalescent patients and controls (Figure 3Bvii). The percentages of NKp44^+^ NKT-like cells were higher in acute patients than in controls [acute: 35.2 (0−96.9) vs. controls: 19.5 (1.6−33.1), *p* = 0.001]. The percentage of NKp44^+^ NKT-like cells was higher in acute than in convalescent patients [acute: 35.2 (0−96.9) vs. convalescent: 19.6 (0.1−96), *p* = 0.035] (Figure 3Aviii). Higher percentages of NKT-like cells bearing CD244^+^ receptors were observed in both patient categories [acute: 40.7 (0−100), convalescent: 25.9 (0−90.5) vs. controls: 0.9 (0−3.3), *p* < 0.001 for each] (Figure 3Axi). The MFI of NKp44^+^ and CD244^+^ NKT-like cells were high in patient categories than in controls (Figures 3Bviii,xi). Among the patient categories, MFI of NKp44^+^ NKT-like cells was high in acute than in controls (Figure 3Bviii). Percentage of NKp46^+^ NKT-like cells was comparable between studied categories (Figure 3Aix) (data not shown); however, lower MFI was observed in the patient categories than in controls (Figure 3Bix). Lower percentages of NKG2D^+^ NKT-like cells were observed in both patient categories compared to controls [acute: 39.2 (4.9−93.5), convalescent: 36.2 (9.6−95.8) vs. controls: 81.3 (33.8−98.3), *p* < 0.001 for each] (Figure 3Ax). In consistent with the percentage of NKG2D^+^ NK cells, MFI was also lower in patient categories than in controls (Figure 3Bx). The percentages of NKT-like cells bearing CD161^+^ receptors were higher in acute patients than in controls [acute: 45.6 (19.7−68.6) vs. controls: 31.6 (12–72.2), *p* = 0.018]. CD161^+^ NK/NKT-like cells were not assessed in the convalescent group (Figures 3Avi,xii). The MFI of CD161^+^ NKT-like cells was higher in acute patients, whereas MFI of CD161^+^ NK cells was comparable (Figures 3Bvi,xii). The percentages of CD94^+^ and NKG2A^+^ NK/NKT-like cells in the acute and control groups were comparable (data not shown). The gating strategy used for NKR enumeration is shown in Figure 2iv.

### Functional analysis of NK and NKT-like cells in acute patients

#### Effective cytotoxicity

**Figure 4:**
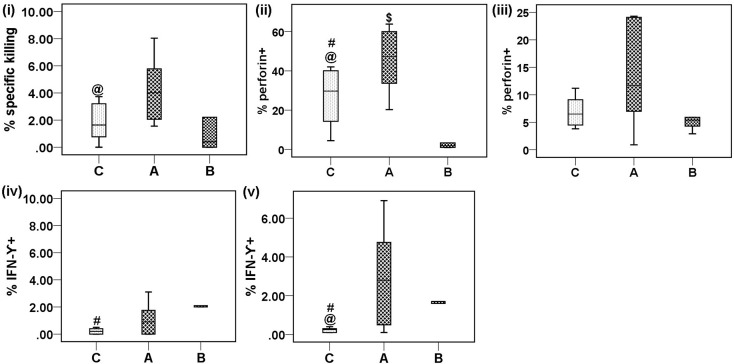
**Cytotoxic potential, intracellular perforin and cytokine expression of NK/NKT-like cells against target cells (K562)**. PBMCs (effector) were isolated from the whole blood of eight control subjects, eight acute patients, and six convalescent patients. PBMCs were co-cultured with K562 cells (target) at an effector/target ratio of 10:1 for 6 h. Box plots show, (i) % specific killing against target cells, (ii) perforin expression by NK cells, (iii) perforin expression by NKT-like cells, (iv) IFN-γ by NK cells, and (v) IFN-γ by NKT-like cells. Mann–Whitney U-test/Kolmogorov–Smirnov test was used for intergroup comparison. *p* < 0.05 is considered significant.

To assess the correlation between alterations in expression of activation receptors on NK/NKT-like cells and their functionality, we performed an effector/target cell based assay. PBMCs showed significantly higher cytotoxicity activity in acute CHIK patients than in controls [acute: 4.1 (1.55−8.04) vs. controls: 1.8 (0−3.73), *p* = 0.027] (Figure 4i).

#### Degranulation assay and perforin expression

An effector/target cell based flow cytometry assay was carried out to assess the expression of CD107a and perforin on NK/NKT-like cells. A higher percentage of perforin^+^ NK cells was observed in the acute CHIK group than in the control and convalescent groups [acute: 45.4 (20.3−63.8), vs. control: 26.4 (4.4−42) and vs. convalescent: 12 (1−63), *p* = 0.046 and 0.039 respectively] (Figure 4ii). A lower percentage of perforin^+^ NK cells was observed in the convalescent group than in the control group [control: 26.4 (4.4−42) vs. convalescent: 12 (1−63), *p* = 0.023] (Figure 4ii). The percentage of perforin+ NKT-like cells was comparable among the groups (data not shown). CD107a expression on NK and NKT-like cells was comparable among the studied groups (Figure 4iii) (data not shown). However, the MFI of CD107a^+^ NK cells from acute CHIK patients was higher than in controls (Supplementary Figure 1).

#### Intracellular cytokines (IFN-γ and TNF-α)

To evaluate the impact of CHIKV infection on the cytokine production capacity of NK/NKT-like cells, we performed intracellular cytokine staining and the percentages of positive cells were enumerated using flow cytometry. A Kolmogorov–Smirnov test showed that IFN-γ expression was higher on NKT-like cells of acute CHIK patients than in controls [acute: 2.8 (0.1-6.9) vs. controls: 0.2 (0.1−0.4), *p* = 0.042)] (Figure 4v). Higher expression of IFN-γ was also observed on NK [convalescent: 3.1 (1.3−8.2) vs. controls: 0.2 (0.1-0.5), *p* = 0.009)] and NKT-like [convalescent: 2.1 (0.8−4.8) vs. controls: 0.2 (0.1−0.4), *p* = 0.009)] cells of the convalescent group compared to the control group (Figures 4iv,v). TNF-α expression was comparable between all groups (data not shown).

### Peripheral T lymphocyte subsets in CHIK patients

**Figure 5:**
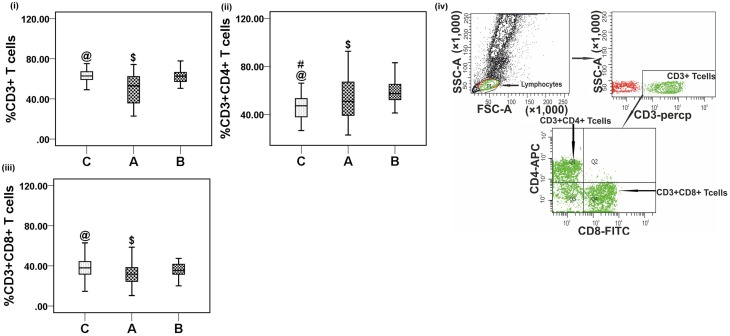
**Distribution of CD3^+^, CD3^+^CD4^+^ and CD3^+^CD8^+^ T cells**. Percentages of the lymphocytes mentioned were determined from the whole blood of 36 control (represented by “C”) subjects, 45 acute (represented by “A”), and 31 convalescent (represented by “B”) patients. Box plots show the percetage of (i) CD3^+^ T cells, (ii) CD3^+^CD4^+^ T cells, and (iii) CD3^+^CD8^+^ T cells. Kolmogorov–Smirnov test was used for intergroup comparison. *p* < 0.05 is considered significant. (iv) Plots showing the strategy used for gating lymphocytes, CD3^+^ T, CD3^+^CD4^+^ T, and CD3^+^CD8^+^ T cells. Lymphocytes are gated in FSC vs. SSC plot, CD3^+^ T cells were gated within the lymphocytes population, CD3^+^CD4^+^ and CD3^+^CD8^+^ T cells were gated on CD3^+^ T cells. Contour plot shows CD3^+^ T cell gating, upper left quadrant (Q1) of the contour plot shows CD3^+^CD4^+^ T cells and lower right quadrant (Q4) shows CD3^+^CD8^+^ T cells.

To investigate the impact of CHIKV infection on the phenotypes of T cells, percentages of CD3^+^, CD3^+^CD4^+^, and CD3^+^CD8^+^ T cells were enumerated using flow cytometry of samples from acute and convalescent patients. Lower percentages of both CD3^+^and CD3^+^CD8^+^ T cells were observed in acute patients than in controls [CD3^+^: acute, 48.7 (22.9−74.3) vs. controls, 63.4 (49.1−79.3), *p* < 0.001 and CD3^+^CD8^+^: acute, 32.3 (10.4−98.2) vs. controls, 38.01 (14.6−63), *p* = 0.013] (Figures 5i,iii). A lower absolute count of both CD3^+^ [acute: 831 (250−1639) and controls: 1519 (1157−1822), per mm^3^] and CD3^+^CD8^+^ T cells [acute: 471 (226−669) and controls: 934 (724−1180), per mm^3^] was observed in acute CHIK patients than in controls (Supplementary Table 1). The percentage of CD3^+^CD4^+^T cells was higher in both patient groups than in controls [CD3^+^CD4^+^: acute, 52.2 (23−92.6) vs. controls, 46.1 (26.7−66.1), *p* = 0.02 and convalescent, 58.8 (41.4−86.6) vs. controls, 46.1 (26.7−66.1), *p* < 0.001] (Figure 5ii). The percentages of CD3^+^, CD3^+^CD4^+^, and CD3^+^CD8^+^T cells were higher in convalescent than in acute [CD3^+^: acute, 48.7 (22.9−74.3) vs. convalescent, 60.7 (10.−77.8), *p* = 0.003; CD3^+^CD4^+^: acute, 52.2 (23−92.6) vs. convalescent, 58.8 (41.4−86.6), *p* = 0.032 and CD3^+^CD8^+^: acute, 32.3 (10.4−98.2) vs. convalescent: [35.3 (2.72−69.7), *p* = 0.032] (Figures 5i–iii). Gating strategies for CD3^+^, CD3^+^CD4^+^, and CD3^+^CD8^+^ T cell enumeration are shown in Figure 5iv. The data are represented as means (range).

Representative figures for T lymphocyte subsets proportion are depicted in Figure 1i.

### CHIKV-specific IFN-γ release in elispot

**Figure 6:**
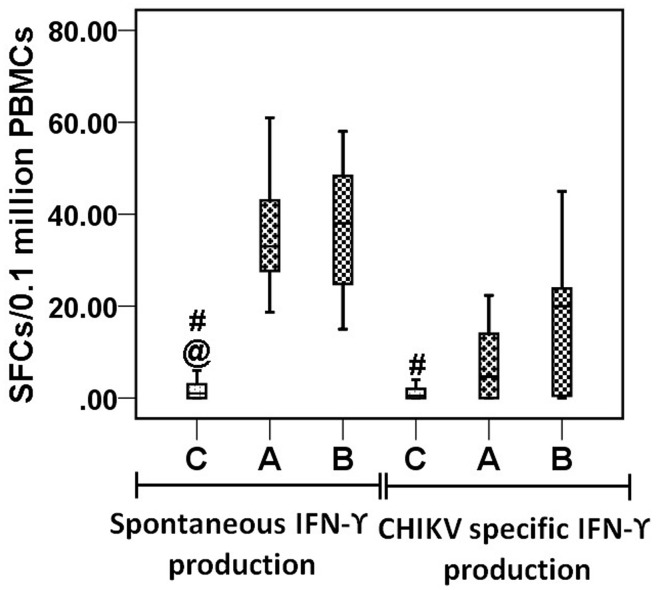
**IFN-γ responses by ELISPOT**. Box plots are showing IFN-γ responses by PBMCs from 18 control subjects, 13 acute, and 15 convalescent patients, stimulated with CHIK antigen (inactivated CHIKV 10 μg/10^6^ PBMCs). Y axis is showing spot forming cells (SFCs) per 10^5^ million cells. Plot shows IFN-γ responses by unstimulated PBMCs before subtraction from stimulated PBMCs well (spontaneous IFN-γ production) and after subtraction of spots in unstimulated PBMCs well from stimulated well (CHIKV specific IFN-γ production). Non-parametric Kolmogorov–Smirnov test was used for intergroup comparison. *p* < 0.05 is considered significant.

To determine the CHIKV-specific IFN-γ response, we performed an ELISPOT assay using whole CHIKV virus particles as antigens. In the control group (*n* = 18), the IFN-γ responses in unstimulated, CHIKV-stimulated, and PHA-stimulated cells were 1 (0−6), 2 (0−13), and 113 (25−248) SFCs/10^5^ cells, respectively. In the acute patient group (*n* = 13), the IFN-γ responses in unstimulated, CHIKV-stimulated, and PHA-stimulated cells were 36 (18−61), 41 (22−70), and 165 (52−390), respectively. In the convalescent group (*n* = 15), the corresponding figures were 36 (15−58), 53 (20−148), and 152 (31−339). IFN-γ responses in unstimulated cells of both patient groups were higher than in controls (Figure 6). IFN-γ spots in the antigen-stimulated wells for the control, acute, and convalescent groups were 1 (0−8), 7 (0−22), and 20 (0−107), respectively, after subtracting the average number of spots in unstimulated wells from antigen-stimulated wells in each category. CHIKV-specific IFN-γ responses were higher in the convalescent group than in the control group (*p* = 0.016) (Figure 6).

## Discussion

The type I interferon production and NK cell expression in patients in the early phase of CHIKV infection indicated the involvement of innate immune responses to CHIKV infection (Her et al., 2010; Petitdemange et al., 2011). Therefore, a detailed investigation of NK and NKT-like cell activities was undertaken in a transverse study from a CHIK outbreak, to explore the interplay between innate and adaptive responses in CHIKV infection.

The lower percentages of CD3^+^CD8^+^ and CD3^+^ T cells observed in the acute patient group were concordant with the reported decrease in the percentage of CD3^+^ T cells in CHIKV-infected patients and supports the hypothesis of infection-associated profound T cell lymphopenia (Petitdemange et al., 2011). The down regulation of both T cell populations could be a transient phenomenon, as shown by the comparable values observed in controls and convalescent patients. However, there are reports of high levels of CD8^+^ T cells in acute patients (Hoarau et al., 2010; Wauquier et al., 2011). The difference in the results may be related to the large differences in sample size/the differences in the cluster of differentiation (CD) markers used/differences in the immune response of the two geographically different populations studied. A study using a mouse model indicated that CD8^+^ T cells play no role in the antiviral response or pathology of CHIKV infection. The higher levels of CD3^+^CD4^+^ T cells observed in the current patient population could account for the joint swelling observed, as reported in a study using a mouse model (Teo et al., 2013).

The positive correlation observed between the percentage of NK cells and the CHIKV load is concordant with the observations of Petitdemange et al. suggesting the involvement of NK cells from the inception of CHIKV infection (Petitdemange et al., 2011).

The function of NK cells is tightly orchestrated by a balance between signals derived from inhibitory and activation receptors (Cooper et al., 2001; De Maria et al., 2011). Activation receptors, i.e., NKG2C, NKG2D, and DNAM-1, and natural cytotoxicity receptors, i.e., NKp46, NKp44, and NKp30, along with various co-stimulatory receptors are involved in human NK cell activation (Moretta et al., 2001; Lanier, 2005). Reports that higher expression of the activation receptors NKp30, NKp44, NKp46, and CD244 and lower levels of the inhibitory receptors NKG2A and CD94 on NK cells produce an effective cytotoxic response are concordant with the results observed in the acute patient population studied here (Biron et al., 1999; De Maria et al., 2001; Lanier, 2005). Corroborating these data, higher MFI of CD107a^+^ NK cells in the acute patients than in controls suggests that, the degranulation capacity per NK cell in the acute CHIKV patients is higher. In a similar line, Srivastava et al. have demonstrated comparable cytotoxic activity against K562 cells in PBMCs in acute Hepatitis E patients regardless of lower NK cells percentage. This phenomenon has been attributed to a greater cytotoxic potential per cell of the PBMCs from acute patients (Srivastava et al., 2008). Higher expression of NKp44 and lower levels of NKG2A, NKp30, and NKp46, associated with cytotoxic NK cells, has been reported in Gabonese CHIK patients (Petitdemange et al., 2011). Hershkovitz et al. demonstrated a direct protein-protein interaction between *Flavivirus* E protein and NKp44 that triggers the secretion of granules contained in NK cells, suggesting NKp44-mediated NK cytotoxic activity (Hershkovitz et al., 2009). Similarly, the higher percentage of NKp44^+^ NK/NKT-like cells and the higher cytotoxic potential of NK cells observed in acute CHIK patients suggest that NKp44-CHIKV protein interaction studies would be useful. The higher expression of NKp44^+^ NK cells observed in CHIK patients could be indicative of the activation of NK cells in CHIKV infection, irrespective of their replicative status. NKp46^+^ NK cells in acute patients might modulate cytotoxicity, as reported in other viral infections (Mandelboim et al., 2001; Krämer et al., 2012). A lower level of CD244^+^ (2B4) expression has been correlated with impaired cytotoxic and IFN-γ-producing activities of NK cells (Marcoe et al., 2012). Reverse observation of the current data indicates CD244^+^ as a mediator for NK/NKT-like cell activation. CD244^+^-mediated signaling can contribute both positively and negatively to viral clearance and immune-mediated disease (Marcoe et al., 2012). An understanding of how CD244 regulates both effector cell function and NK/T cell crosstalk may offer opportunities to define the roles of subsets of NK cells in early CHIKV infection. Lower expression of NKG2D^+^ NK/NKT-like cells observed here and in herpes virus infection suggests that NKG2D^+^ cells contribute little to activation status (Jonjic et al., 2008; Muntasell et al., 2010). However, Petitdemange et al. have shown a functional NK cells in spite of comparable NKG2D expression on NK cells in CHIK patients (Petitdemange et al., 2011). The higher expression of CD161^+^ NKT-like cells observed in acute patients suggests the need for further exploration. The elevation in activation receptors observed in the current patient population could be responsible for NK cell proliferation and might play a role in NK cell maintenance during infection and may deliver direct antiviral effects, as previously reported (Vidal et al., 2011).

The functionality of NK cells requires the differential engagement of cell surface receptors in combination with activities mediated by pro-inflammatory cytokines. The higher IFN-γ expression observed on NKT-like cells of acute CHIK patients leads us to hypothesize that NKT-like cells play an immune regulatory role toward the cytotoxicity of NK cells. The IFN-γ release observed in the ELISPOT assay and the IFN-γ expression observed on both NK/NKT-like cells in the convalescent stage are suggestive of the key roles played by these cells in the development of an effective adaptive immune response. In contrast to the reported role of perforin in an alphavirus-infected mouse model, the higher perforin expression observed on NK cells of acute patients suggested that cytotoxicity could be perforin-mediated phenomena (Kägi and Hengartner, 1996).

It may be a coincidence that our current study population from the recent CHIK outbreak contained more male than female individuals. Further, statistical analyses of each assay parameter following separation by gender were comparable to the pooled results, suggesting that the data were not influenced by the gender ratio.

Finally, we showed that, during acute CHIKV infection, NK cells are cytotoxic (perforin), and that NKT-like cells elicited an effector response (IFN-γ expression) followed by an adaptive response (IFN-γ release in the ELISPOT assay and IFN-γ expression on NK/NKT-like cells) in the convalescent phase. The heightened cytotoxicity and comparable effector functions of NK cells suggested a dichotomy of NK cells in acute CHIKV infection. The present study elucidates the potential role of activation receptors on cytotoxic NK cells in influencing the pathogenesis of early CHIKV infection. Further, study of the molecular mechanisms that limit viral dissemination/establishment of chronic disease will aid in understanding how NK/NKT-like cells control CHIKV infection.

## Author contributions

AT and ST conceived the work. ST and RD conducted and interpreted the laboratory work. ST and AT wrote the manuscript. All authors contributed to, and read and approved the final manuscript. AT is guarantor for the paper.

### Conflict of interest statement

The authors declare that the research was conducted in the absence of any commercial or financial relationships that could be construed as a potential conflict of interest.

## Supplementary material

Supplementary Figure 1 **CD107a expression by NK and NKT-like cells against target (K562) cells**. PBMCs (effector) were isolated from the whole blood of 8 control subjects, 8 acute patients, and 6 convalescent patients. PBMCs were co-cultured with target cells at an effector/target ratio of 10:1 for 6 h. @ denotes A (acute) vs. C(control), # denotes B (convalescent) vs. C (control) and $ denotes A (acute) vs.C (convalescent). **(A)** Box plots are showing the percentage of CD107a^+^ (i) NK cells and (ii) NKT-like cells. **(B)** Box plots are showing the mean fluorescence intensity (MFI) of CD107a^+^ (i) NK cells and (ii) NKT-like cells. Kolmogorov–Smirnov test was used for intergroup comparison. *p* < 0.05 is considered significant.

Supplementary Table 1 **Absolute count of CD3^+^, CD3^+^CD4^+^, CD3^+^CD8^+^, CD3^−^CD56^+^, and CD3^+^CD56^+^ cells from the whole blood of CHIKV infected patients**.
