# Supplementary figures and images for: Phenotypic and functional analyses of NK and NKT-like populations during the early stages of chikungunya infection
Source: Front Microbiol. 2015 Sep 1;6:895. doi: 10.3389/fmicb.2015.00895 (PMC4555083; doi:10.3389/fmicb.2015.00895)

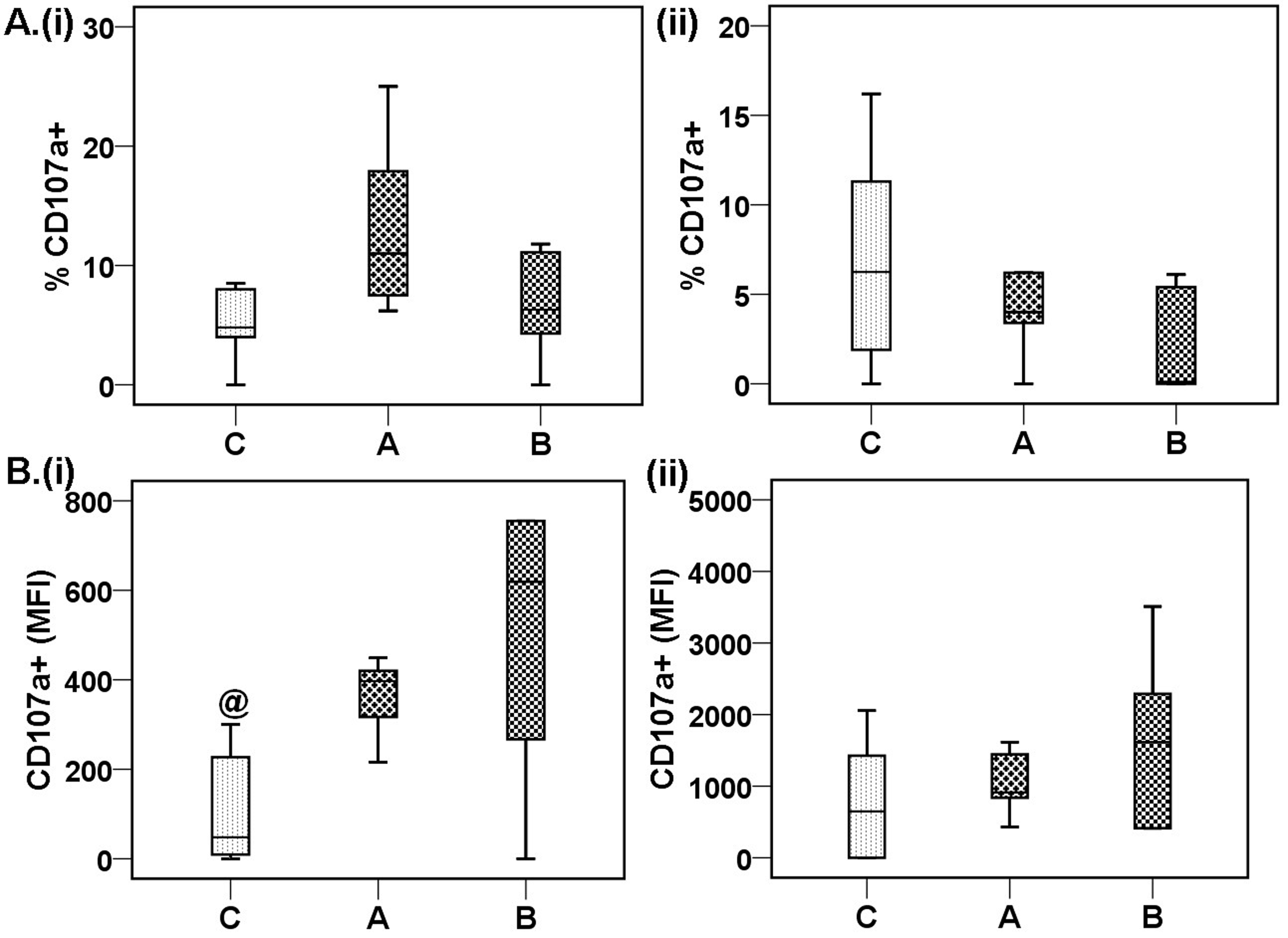

Supplement: Supplementary Figure 1 — CD107a expression by NK and NKT-like cells against target (K562) cells. PBMCs (effector) were isolated from the whole blood of 8 control subjects, 8 acute patients, and 6 convalescent patients. PBMCs were co-cultured with target cells at an effector/target ratio of 10:1 for 6 h. @ denotes A (acute) vs. C(control), # denotes B (convalescent) vs. C (control) and $ denotes A (acute) vs.C (convalescent). (A) Box plots are showing the percentage of CD107a+ (i) NK cells and (ii) NKT-like cells. (B) Box plots are showing the mean fluorescence intensity (MFI) of CD107a+ (i) NK cells and (ii) NKT-like cells. Kolmogorov–Smirnov test was used for intergroup comparison. p < 0.05 is considered significant. [file Image1.TIF]
