# Supplementary material for: Phenotypic and functional analyses of NK and NKT-like populations during the early stages of chikungunya infection
Source: Front Microbiol. 2015 Sep 1;6:895. doi: 10.3389/fmicb.2015.00895 (PMC4555083; doi:10.3389/fmicb.2015.00895)
Supplement: Supplementary Table 1 — Absolute count of CD3+, CD3+CD4+, CD3+CD8+, CD3−CD56+, and CD3+CD56+ cells from the whole blood of CHIKV infected patients. [file Table1.DOCX]

**TABLE. 1 Absolute count of CD3^+^, CD3^+^CD4^+^, CD3^+^CD8^+^, CD3^-^CD56^+^ and CD3^+^CD56^+^ cells from the whole blood of CHIKV infected patients.**

| **Categories** | **CD3^+^ cells/ mm^3^of blood** | **CD3^+^CD4^+^ cells/ mm^3^ of blood** | **CD3^+^CD8^+^ cells/ mm^3^ of blood** | **CD3^-^CD56^+^ cells/ mm^3^ of blood** | **CD3^+^CD56^+^ cells/ mm^3^ of blood** |
| --- | --- | --- | --- | --- | --- |
| Acute | 831 (250-1639) | 1266 (622-1645) | 471 (226-669) | 233 (158-270) | 250 (178-406) |
| Control | 1519 (1157-1822) | 1162 (580-1469) | 934 (724-1180) | 175 (101-360) | 148 (36-492) |

Control represents 10 healthy individuals. Data are expressed as mean (range).
